# Complete Genome Sequence of a New *Clostridium* sp. Isolated from Anaerobic Digestion and Biomethanation

**DOI:** 10.1128/MRA.01472-19

**Published:** 2020-01-16

**Authors:** Sarah Hahnke, Christian Abendroth, Javier Pascual, Thomas Langer, Patrice Ramm, Michael Klocke, Olaf Luschnig, Manuel Porcar

**Affiliations:** a Leibniz Institute for Agricultural Engineering and Bioeconomy, Bioengineering, Potsdam, Germany; b University of Oldenburg, Department of Human Medicine, Oldenburg, Germany; c Robert Boyle Institut e.V., Jena, Germany; d Institute of Waste Management and Circular Economy, Technische Universität Dresden, Pirna, Germany; e Darwin Bioprospecting Excellence, S.L., Paterna, Valencia, Spain; f Institute of Agricultural and Urban Ecological Projects affiliated to Berlin Humboldt University, Berlin, Germany; g Bio H2 Energy GmbH, Jena, Germany; h Institute for Integrative Systems Biology, University of Valencia-CSIC, Paterna, Valencia, Spain; Indiana University, Bloomington

## Abstract

Here, we present the genome sequence and annotation of the bacterial strain HV4-5-A1G, a potentially new *Clostridium* species. Based on its genomic data, this strain may act as a keystone microorganism in the hydrolysis of complex polymers, as well as in the different acidogenesis and acetogenesis steps during anaerobic digestion.

## ANNOUNCEMENT

We present the genome sequence and annotation of the bacterial strain HV4-5-A1G, which was isolated from the acidification stage of a mesophilic two-stage laboratory-scale leach bed system, which worked as described by Abendroth et al. (1). Freshly cut grass was taken from a meadow in Jena, Germany (50°51ʹ55.4ʺN, 11°35ʹ56.1ʺE), as the sole substrate. Isolation of the strain was performed after the diluted hydrolysate was reincubated with microcrystalline cellulose as the sole carbon source. After incubation, the hydrolysate was diluted 10-fold, plated on reinforced clostridial agar (Oxoid Ltd.), and cultivated under anoxic conditions at 37°C. For purification, colonies were picked and restreaked several times. Comparison of the almost complete 16S rRNA gene sequence of the strain with those in the NCBI database suggested that the strain belongs to a new species, as the closest type strain, Clostridium luticellarii FW431^T^, showed only 97.3% sequence similarity (2); therefore, this strain was chosen for genome sequencing. After cultivation in brain heart infusion broth (Carl Roth GmbH & Co. KG) supplemented with yeast extract, DNA was extracted and purified using the Gentra Puregene Yeast/Bact. kit (Qiagen). For subsequent purification of the DNA, the NucleoSpin genomic DNA cleanup kit (Macherey-Nagel) was used. We prepared a Nextera XT library from total genomic DNA and sequenced it using the Illumina NextSeq 500 platform (150-bp paired-end reads). Raw reads were filtered (Q score, >20; minimum length, >50 nucleotides [nt]) with BBTools v37.10, yielding 26.98 million paired-end sequences with a mean Q value of 33.41. Genome assembly was conducted with SPAdes 3.13.0 (3). A total of 287 contigs with a length of ≥300 nt were obtained, covering a total genome size of 3,834,313 nt, with an estimated GC content of 34.17%. The largest contig was 93,925 nt, and the *N*_50_ value was 30,423 nt. Default parameters were used for all software unless otherwise specified.

The assembled sequences were annotated using NCBI Prokaryotic Genome Annotation Pipeline 4.9 (4) and the RAST toolkit (5) implemented in the genome annotation service in PATRIC (6). The genome of strain HV4-5-A1G harbors 3,778 genes, including 3,689 open reading frames, 92 pseudogenes, 5 5S rRNAs (all complete), 3 16S rRNAs (partial), 9 23S rRNAs (partial), 67 tRNAs, and 5 noncoding RNAs. A total of 12 CRISPR arrays were identified.

The genome harbors nine different glycoside hydrolases, including two cellulases (7). Furthermore, genes for NAD^+^-dependent ethanol dehydrogenase and l-lactate dehydrogenase indicate the capability for alcoholic fermentation and lactate formation. Moreover, HV4-5-A1G is potentially able to conduct the fermentation of lactate to acetate and propionate via methylmalonyl-coenzyme A (CoA) with the enzymes methylmalonyl-CoA mutase and methylmalonyl-CoA epimerase, as well as the conversion of acetyl-CoA to acetate with acetate kinase and phosphate acetyltransferase. Therefore, strain HV4-5-A1G may have a key role in the hydrolysis of complex polymers and in the different acidogenesis and acetogenesis steps during anaerobic digestion of organic matter.

### Data availability.

Strain HV4-5-A1G was deposited in the German Collection of Microorganisms and Cell Cultures under the designation DSM 104276. This whole-genome sequencing (WGS) project was deposited in DDBJ/ENA/GenBank under accession number VXJK00000000. The version described in this paper is version VXJK01000000. Raw sequence reads were deposited in the SRA under accession number SRR10177393. The WGS and SRA records are associated with BioProject number PRJNA565868.

## References

[1] Abendroth C, Hahnke S, Simeonov C, Klocke M, Casani-Miravalls M, Ramm P, Bürger C, Luschnig O, Porcar M 2018 Microbial communities involved in biogas production exhibit high resilience to heat shocks. Bioresour Technol 249:1074–1079. doi:10.1016/j.biortech.2017.10.093. 29146311

[2] Richter M, Rosselló-Móra R, Oliver Glöckner F, Peplies J 2016 JSpeciesWS: a Web server for prokaryotic species circumscription based on pairwise genome comparison. Bioinformatics 32:929–931. doi:10.1093/bioinformatics/btv681. 26576653 PMC5939971

[3] Bankevich A, Nurk S, Antipov D, Gurevich AA, Dvorkin M, Kulikov AS, Lesin VM, Nikolenko SI, Pham S, Prjibelski AD, Pyshkin AV, Sirotkin AV, Vyahhi N, Tesler G, Alekseyev MA, Pevzner PA 2012 SPAdes: a new genome assembly algorithm and its applications to single-cell sequencing. J Comput Biol 19:455–477. doi:10.1089/cmb.2012.0021. 22506599 PMC3342519

[4] Tatusova T, DiCuccio M, Badretdin A, Chetvernin V, Nawrocki EP, Zaslavsky L, Lomsadze A, Pruitt KD, Borodovsky M, Ostell J 2016 NCBI Prokaryotic Genome Annotation Pipeline. Nucleic Acids Res 44:6614–6624. doi:10.1093/nar/gkw569. 27342282 PMC5001611

[5] Brettin T, Davis JJ, Disz T, Edwards RA, Gerdes S, Olsen GJ, Olson R, Overbeek R, Parrello B, Pusch GD, Shukla M, Thomason JA, Stevens R, Vonstein V, Wattam AR, Xia F 2015 RASTtk: a modular and extensible implementation of the RAST algorithm for building custom annotation pipelines and annotating batches of genomes. Sci Rep 5:8365. doi:10.1038/srep08365. 25666585 PMC4322359

[6] Wattam AR, Davis JJ, Assaf R, Boisvert S, Brettin T, Bun C, Conrad N, Dietrich EM, Disz T, Gabbard JL, Gerdes S, Henry CS, Kenyon RW, Machi D, Mao C, Nordberg EK, Olsen GJ, Murphy-Olson DE, Olson R, Overbeek R, Parrello B, Pusch GD, Shukla M, Vonstein V, Warren A, Xia F, Yoo H, Stevens RL 2017 Improvements to PATRIC, the all-bacterial bioinformatics database and analysis resource center. Nucleic Acids Res 45:D535–D542. doi:10.1093/nar/gkw1017. 27899627 PMC5210524

[7] Lombard V, Golaconda Ramulu H, Drula E, Coutinho PM, Henrissat B 2014 The Carbohydrate-Active Enzymes database (CAZy) in 2013. Nucleic Acids Res 42:D490–D495. doi:10.1093/nar/gkt1178. 24270786 PMC3965031

